# Bayesian spatial modelling of tuberculosis-HIV co-infection in Ethiopia

**DOI:** 10.1371/journal.pone.0283334

**Published:** 2023-03-23

**Authors:** Leta Lencha Gemechu, Legesse Kassa Debusho

**Affiliations:** Department of Statistics, College of Science, Engineering and Technology, University of South Africa, Johannesburg, South Africa; Bangladesh Agricultural University, BANGLADESH

## Abstract

An in-depth analysis of the epidemiological patterns of TB/HIV co-infection is essential since it helps to target high-risk areas with effective control measures. The main objective of this study was to assess the spatial clustering of TB/HIV co-infection prevalence in Ethiopia for the year 2018 using district-level aggregated TB and HIV data obtained from the Ethiopian Federal Ministry of Health. The global Moran’s index, Getis-Ord $G_{i}^{*}$ local statistic, and Bayesian spatial modeling techniques were applied to analyse the data. The result of the study shows that TB among people living with HIV (PLHIV) and HIV among TB patients prevalence were geographically heterogeneous. The highest prevalence of TB among PLHIV in 2018 was reported in the Gambella region (1.44%). The overall prevalence of TB among PLHIV in Ethiopia in the same year was 0.38% while the prevalence of HIV among TB patients was 6.88%. Both district-level prevalences of HIV among TB patients and TB among PLHIV were positively spatially autocorrelated, but the latter was not statistically significant. The local indicators of spatial analysis using the Getis-Ord statistic also identified hot-spots districts for both types of TB/HIV co-infection data. The results of Bayesian spatial logistic regression with spatially structured and unstructured random effects using the Besag, York, and Mollié prior showed that not all the heterogeneities in the prevalence of HIV among TB patients and TB among PLHIV were explained by the spatially structured random effects. This study expanded knowledge about the spatial clustering of TB among PLHIV and HIV among TB patients in Ethiopia at the district level in 2018. The findings provide information to health policymakers in the country to plan geographically targeted and integrated interventions to jointly control TB and HIV.

## Background

Human immunodeficiency virus (HIV) and tuberculosis (TB) are major global public health concerns. TB is the single infectious disease that takes more lives each year, ranking above HIV until the coronavirus (COVID-19) pandemic spread worldwide [1]. TB and HIV diseases are epidemiologically associated [2]. Observed co-dynamics suggest that the two diseases are directly related at the population level [3] and also within the host [4].

Although TB is a preventable and treatable disease, it is the leading cause of death among people living with HIV (PLHIV) claiming millions of lives each year [5]. The emergence of the HIV pandemic has also had a major impact on TB incidence rates [6]. HIV affects TB epidemiology by altering the natural course of infection and increasing the risk of latent TB infection (LTBI) reactivation [7]. According to WHO, the risk of developing active TB is estimated to be 20 times greater in PLHIV than in people who are HIV-negative [8]. Sub-Saharan Africa is the hardest-hit region as it is home to 70% of all people living with TB/HIV co-infection in the world [9]. In 2018, on average PLHIV were 19 times more likely to fall ill with TB than those without HIV. In this year, an estimated 862,000 PLHIV worldwide fell ill with TB, of which 251,000 people (about one-third of total AIDS deaths) died from HIV-associated TB cases. [10]. In 2019, there were an estimated 815,000 global TB incident cases among PLHIV, 55% of which were diagnosed. In addition, there were an estimated 210,000 TB-related deaths among PLHIV, about a 63% reduction compared to 2010, where TB-related death was 570,000 among PLHIV [11].

In 2016, an estimated 16,000 PLHIV had or were infected with TB in Ethiopia. During this year, the routine National Tuberculosis Program (NTP) reported that 8,625 (about 54%) were receiving co-treatment. A study in Ethiopia also suggests that TB incidence rate is high in areas where HIV is highly prevalent [12]. Based on the WHO recommendation, the integrated TB and HIV collaborative activities were updated in 2012, and aimed at reducing the burden of both HIV among TB patients and TB among PLHIV. The Ethiopian guidelines recommend functional TB/HIV collaborative mechanisms at all levels, which include national, regional, sub-regional or district and health service facility levels. Studies on the prevalence of TB/HIV co-infection have shown that the co-infection varies widely in Ethiopia (see e.g. [12, 13]) and has geographical clustering [14]. Other studies, e.g. [15, 16] have reported the epidemiology of TB/HIV co-infection at the hospital level in Ethiopia. Similar observations have been made in other countries [17, 18]. Additionally, the recent work of Alene et al. [14] justified the spatial distribution of TB/HIV co-infection in Ethiopia at a national level using three-year aggregated data. However, to our knowledge, there is no report on Bayesian spatial modeling of TB/HIV co-infection at least using yearly data at a national level in Ethiopia. In order to design the most effective strategies that can help to reduce the TB and HIV transmission rates, it is essential to have a more in-depth analysis of the epidemiological patterns of TB/HIV co-infection at the district level. Identifying districts or areas where the burden of TB/HIV co-infection is concentrated may help to identify populations at higher risks of co-infection. Targeting high-risk areas with effective control measures yields good results in controlling the pandemic;TB, HIV and TB/HIV [19]. In addition, knowledge of hot-spots and cold-spots (or high and low burden) areas is required for successful surveillance programs and optimal resource allocation [20]. Therefore, the main aim of this study was to assess the spatial distribution of TB/HIV co-infection in Ethiopia at the district level using the Bayesian approach. The Bayesian spatial modeling allows to incorporate spatial or geographical neighborhood information on the variable of interest. Four different Bayesian spatial models were fitted for the data using different priors and the best model was selected using DIC and WAIC measures.

The rest of the paper is organized as follows. We first describe the data, then discuss the Bayesian spatial modelling giving more emphasis to spatial priors applied in the present study. The results are presented in the Results section. Finally, a discussion of the results and the limitations of the study, and the conclusions are given in the Discussion and the Conclusion sections, respectively.

## Methods

### Study area

**Fig 1:**
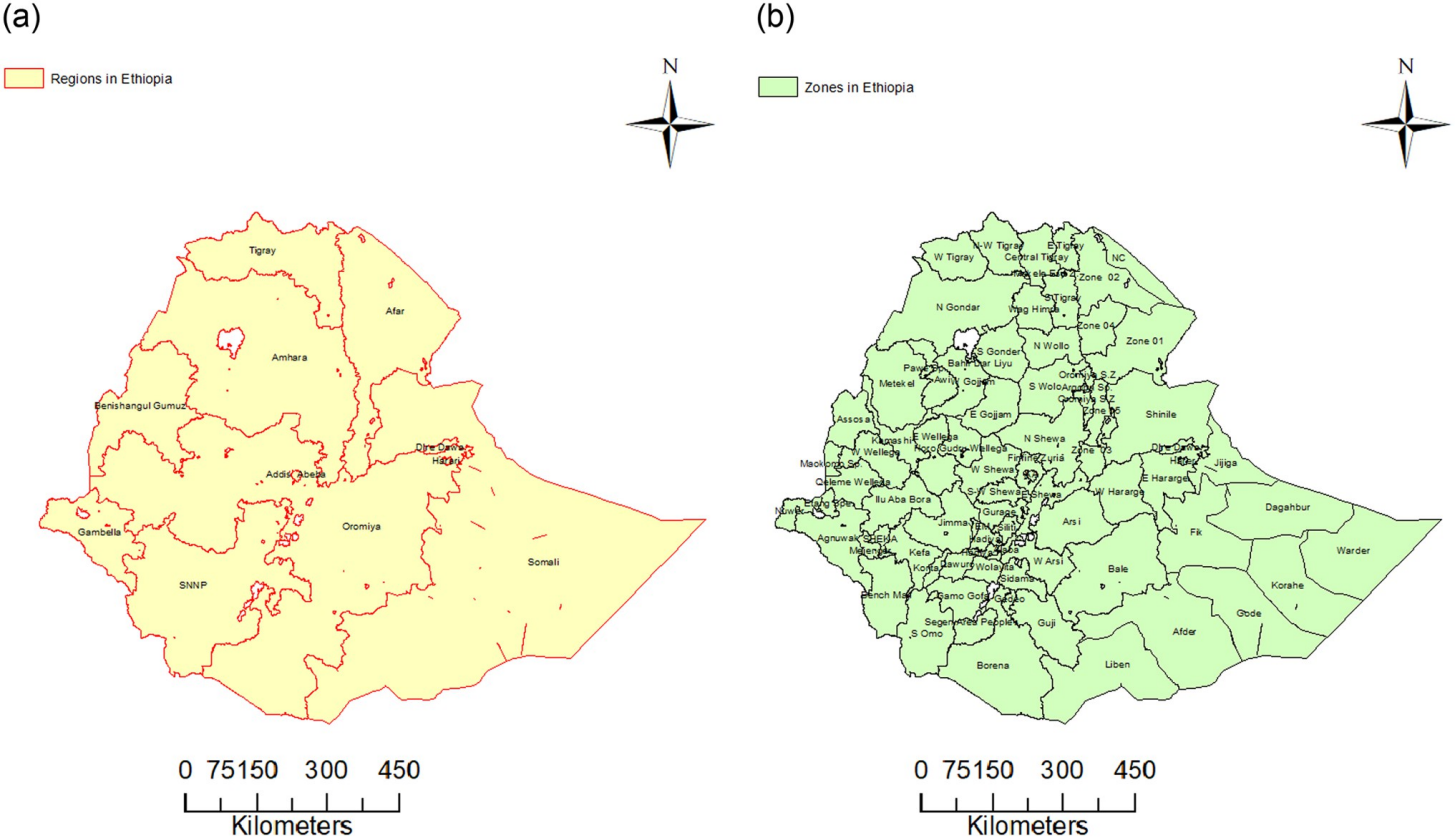
Regions and Zones in Ethiopia. Spatial plot of regions and zones in Ethiopia, where E, N, S, W, N-W, Sp. and Z represent east, noth, south, west, north-west, special and zone, respectively.

Ethiopia is located in the North-Eastern part of Africa. It shares borders with Sudan in the west, Eritrea in the north, Djibouti in the Northeast, Somalia in the east and south east and Kenya in the south. The country occupies an area of approximately 1,127,127 square kilometers. Administratively, before 2020 the Federal Democratic Republic of Ethiopia was divided into nine regional states (Tigray; Afar; Amhara; Oromia; Somali; Benishangul-Gumuz; Southern Nations, Nationalities, and Peoples’ (SNNP); Gambella; and Harari) and two city administrations (Addis Ababa and Dire Dawa). Each regional state was divided into zones, a zone divided into districts (called “woreda”), and a district divided into Kebeles (sub-districts). The spatial plot of the regions and the zones of Ethiopia are displayed in Fig 1. With the devolution of power to regional governments, public service delivery is under the jurisdiction of the regional states. The regional health bureaus are responsible for the administration of public health while the districts are responsible for planning and implementation of the health services. Each district is a clearly delineated administrative and geographical area with a well-defined population. Districts have networks of primary health care units such as health centers, health posts, and a district hospital. Health-related data are compiled at the district level and are reported to the Zone Health Department, Region Health Bureau, and then to the Federal Ministry of Health [21].

### Data sources

The data used in this study was district-level aggregated data of TB and HIV co-infection cases of year 2018. The data includes the total number of TB patients enrolled in the national TB program who tested for HIV, TB patients with HIV positive test results, TB patients with HIV negative test results, HIV positive clients who were screened for TB, HIV positive patients with active TB, and HIV positive patients with no TB. The data were compiled and reported by the district Health Office to the Federal Ministry of Health (FMoH) through the Health Management Information System (HMIS) quarterly [22, 23]. In this study, the data were used to investigate the spatial clustering of TB/HIV co-infection in Ethiopia at the district level. Geographical coordinates with district mapping shape files prepared by the Central Statistics Agency (CSA) of Ethiopia was also obtained from FMoH. The response variables of interest in this study were the prevalence of HIV among TB patients and the prevalence of TB among PLHIV, and their values were calculated from the above-described raw data.

### Statistical analyses

In this paper, spatial analysis was used to identify geographical clustering of the observed prevalence of HIV among TB patients, *r*_(*h*)*i*_, calculated as the proportion of the number of TB patients with HIV positive test results in a district *m*_(*h*)*i*_ to the total number of TB patients who were tested for HIV in the same district *n*_(*t*)*i*_. Similarly, the observed prevalence of TB among PLHIV, *r*_(*t*)*i*_ was defined as the proportion of the number of the TB patients among PLHIV in a district *m*_(*t*)*i*_ to the total number of HIV patients in the same district enrolled in HIV care facilities who were screened for TB during their visit *n*_(*h*)*i*_. Two TB/HIV co-infection prevalences were spatially analyzed separately in order to assess the spatial patterns of the two types of co-infections at the district level in 2018. In the spatial analyses, the district geographical boundaries were geo-referenced and linked to the district data described in the Data Source section. The choropleth maps were developed for visualization using ArcGIS software version 10.3 [24]. The statistical techniques used in this paper are discussed below.

#### Spatial pattern analysis

The global spatial autocorrelation of TB/HIV co-infection, i.e. TB among PLHIV and HIV among TB patients, was investigated using the univariate global Moran’s *I* [25]. Let *x*_*i*_ and *x*_*j*_ denote the observed values at districts *s*_*i*_ and *s*_*j*_, *i*, *j* = 1, …, *m*, where *m* is the number of districts. Then the global Moran’s Index or Moran’s *I* [25] statistic is defined as

$$\begin{array}{c} I=\frac{\sum_{i=1}^{m}\sum_{j=1}^{m}w_{ij}\left(x_{i}-\bar{x}\right)\left(x_{j}-\bar{x}\right)}{W_{0}\sum_{i=1}^{m}{\left(x_{i}-\bar{x}\right)}^{2}}, \end{array}$$ (1)

where $W_{0}=\sum_{i=1}^{m}\sum_{j=1}^{m}w_{ij}$ and spatial weight *w*_*ij*_ is chosen according to the spatial relationship between districts of *x*_*i*_ and *x*_*j*_. If the two districts are neighbors, the *w*_*ij*_ = 1; otherwise *w*_*ij*_ = 0. The simplest and most commonly used neighbourhood definition is given by the binary connectivity matrix [26]:

$$w_{ij}=\{\begin{array}{cc} 1 & \text{if}\;\text{districts}\;s_{i}\;\text{and}\;s_{j}\;\text{share}\;\text{a}\;\text{boundary}\\ 0 & \text{otherwise}. \end{array}$$

According this definition, *w*_*ij*_ = *w*_*ji*_ and *w*_*ii*_ = 0, implies no self-correlation of an element with itself, hence the resulting spatial proximity matrix is necessarily symmetric. In expression (1), if the values of neighboring districts are close and (or values are spatially clustered), then *I* is close to 1 which indicates positive spatial autocorrelation. On the other hand, if most of the neighboring districts have different values (or spatially dispersed), then *I* is close to -1 which indicates a negative spatial autocorrelation [26, 27]. The global Moran’s *I* become close to 0 when the deviation values of some pairs of neighbours and others have deviations in the opposite direction( [28], pp. 175).

#### Getis-Ord $G_{i}^{*}$ statistic

The Getis-Ord $G_{i}^{*}$ local statistic is an alternative to the local Moran’s *I* index determining the type of spatial cluster which can be either hot-spot or cold-spot [29]. It is given by

$$\begin{array}{c} G_{i}^{*}=\frac{\sum_{j=1}^{m}w_{ij}x_{j}-\bar{x}\sum_{j=1}^{m}w_{ij}}{S\sqrt{[m\sum_{j=1}^{m}w_{ij}^{2}-{\left(\sum_{j=1}^{m}w_{ij}\right)}^{2}]/(m-1)}}, \end{array}$$ (2)

where *x*_*j*_ is the disease count in district *j*, *w*_*ij*_ is the spatial weight associated with administrative districts *s*_*j*_ and *s*_*i*_, $\bar{x}=\sum_{j=1}^{m}x_{j}/m$ and $S=\sqrt{\frac{\sum_{j=1}^{m}x_{j}^{2}}{m}-{\bar{x}}^{2}}$, i.e. the standard deviation.

Under the approximate normality assumption [29], $G_{i}^{*}$ is assumed to be an estimate of the normal variant [30]. Depending on the choice of confidence level (e.g. 90%, 95%, or 99%) on a map, clusters with a 90 / 95 /99 percent significance level from a two-tailed normal distribution indicate significant clustering, i.e. cold-spots and hot-spots. For a given confidence interval, a hot-spot cluster consists of districts with *z*-scores which are greater than the upper confidence limit, and a cold-spot cluster consists of districts with *z*-scores which are lower than the lower confidence limit.

Unlike the local Moran’s *I* statistic [31], the Getis-Ord $G_{i}^{*}$ statistic computed for each district is a *z*-scores [32], and hence allows for a more direct interpretation of statistical significance. In addition, more than one confidence interval can be plotted on a map. Therefore, in the current study, Getis-Ord $G_{i}^{*}$ statistic was used to identify the hot-spots and cold-spots district clusters with respect to for the prevalence of HIV among TB patients and TB among PLHIV.

### Bayesian spatial models

Spatial data has a property of spatial dependence [33]. A spatial correlation analysis, discussed in the previous section, can be used to check if this property is present for a given data. In the context of this study the Bayesian approach is based on the prior distributional assumptions that are placed on the district-specific parameters of the spatial dependence structure. Four spatial models with Bayesian smoothing conditional autoregressive (CAR) structure [34, 35] and spatially structured random effects were considered for the analysis. A CAR model assumes a form of local spatial continuity so that neighbouring districts have the same residual risks after accounting for the fixed effects [36]. Suppose that for the case of modelling prevalence of HIV among TB patients, *r*_(*h*)*i*_ = *m*_(*h*)*i*_/*n*_(*t*)*i*_ ∼*Binomial*(*p*_(*h*)*i*_, *n*_(*t*)*i*_).

Then a Bayesian spatial smoothing logistic regression model is defined using a logit as

$$\begin{array}{c} logit\left(p_{(h)i}\right)=\beta_{0}+u_{(h)i}+v_{(h)i},\;\;i=1,\ldots ,m, \end{array}$$ (3)

where *m* is the number of districts (804 in this study), *β*_0_ is the intercept, *u*_(*h*)*i*_ is the unstructured random component which assumed to follow a $u_{(h)i}∼N\left(0,\sigma_{u}^{2}\right)$ distribution, *v*_(*h*)*i*_, is the structured spatial random component associated with district *s*_*i*_. In this paper, the structured spatial random component was modelled with the intrinsic conditional autoregressive (ICAR) [34]; proper CAR (pCAR); Besag, York and Mollié (BYM) [34] and Leroux conditional autoregressive (LCAR) [37] models. Similarly, for the case of modelling the prevalence of TB among PLHIV *r*_(*t*)*i*_ ∼ *Binomial*(*p*_(*t*)*i*_, *n*_(*h*)*i*_) and the model is given as

$$\begin{array}{c} logit\left(p_{(t)i}\right)=\beta_{0}+u_{(t)i}+v_{(t)i},\;\;i=1,\ldots ,m. \end{array}$$ (4)

The above prior models are discussed below.

#### The Intrinsic Conditional Autoregressive (ICAR) model

The Intrinsic Conditional Autoregressive (ICAR) model [34] is a prior model which imposes a spatial dependence structure on *v*_(*h*)*i*_ and *v*_(*t*)*i*_ in models (3) and (4). This is done by specifying a conditional normal distribution for the parameter of each district *s*_*i*_ that depends on the parameters of the neighbours of *s*_*i*_. For example, for each *i* = 1, …, *m*:

$$\begin{array}{c} v_{(t)i}|v_{(t)-i},\sigma_{v}^{2},W∼Normal(\frac{\sum_{j\in {△}_{i}}v(t)j}{m_{i}},\frac{\sigma_{v}^{2}}{m_{i}}), \end{array}$$ (5)

where: **v**_(*t*)−*i*_ denotes the set of district-specific parameters excluding *v*_(*t*)*i*_, $\sigma_{v}^{2}$ is an unknown variance parameter which controls the amount of local smoothing for the entire study region [28], i.e. the amount of variation between the spatially structured random effects; △_*i*_ denotes the set of neighbours of district *s*_*i*_ as defined in **W** and *m*_*i*_ is the number of neighbours of district *s*_*i*_ and equals the sum of the *i*th row of **W**. The joint probability distribution of **v**_(**t**)_ has a form (see [38], pp 410–419)

$$\begin{array}{c} P\left(v_{(t)1},\ldots ,v_{(t)m}\right)=P\left(v_{\left(t\right)}\right)\propto \text{exp}\{-\frac{1}{2\sigma_{v}^{2}}v_{\left(t\right)}^{\prime }\left(D_{w}-W\right)v_{\left(t\right)}\}, \end{array}$$ (6)

where **D**_*w*_ is an *m* × *m* diagonal matrix with the diagonal elements equal to the row sums of the **W** matrix, i.e. the *i*th diagonal element of **D**_*w*_ is (**D**)_*ii*_ is equal to $w_{i+}=\sum_{j=1}^{m}w_{ij}$, ∀ *i*, *i* = 1…, *m*.

The conditional distribution (5) is a local dependence structure that leads to a local or neighbourhood smoothing of the area-specific parameters. Given the parameters of all other areas, the conditional mean $\sum_{j\in {△}_{i}}v_{(t)j}/m_{i}$ only depends on the parameters of the neighbouring areas. With a global dependence structure, the conditional mean of *v*_(*t*)*i*_ depends on all other *v*_(*t*)*j*_ with *j* ≠ *i*. In the ICAR model, the variance parameter $\sigma_{v}^{2}$ is the only unknown parameter, and hence to estimate it via the Bayesian approach a prior distribution is required. Since the ICAR model assumes positive spatial autocorrelation on the area-specific parameters, this is to be tested at the exploratory data analysis stage before fitting the ICAR model. In addition, since the joint distribution (6) is improper, i.e. the distribution does not integrate to 1, the ICAR model places a sum-to-zero constraint, i.e. $\sum_{i=1}^{m}v_{(t)i}=0$ on the district-specific parameters **v**_(*t*)_.

#### The Proper CAR (pCAR) model

The ICAR model can converted to a proper CAR model by introducing a multiplicative parameter *ρ* to the spatial weights. This is defined via a set of *m* full conditionals as

$$\begin{array}{c} v_{i}|v_{-i},\sigma_{v}^{2},W∼Normal(\rho \frac{\sum_{j=1}^{m}w_{ij}v_{j}}{w_{i+}},\frac{\sigma_{v}^{2}}{w_{i+}}), \end{array}$$ (7)

or, equivalently, by the joint multivariate distribution given as:

$$\begin{array}{c} P\left(v_{\left(t\right)}\right)\propto \;\text{exp}\;\{-\frac{1}{2\sigma_{v}^{2}}S_{\left(t\right)}^{\prime }\left(D_{w}-\rho W\right)v_{\left(t\right)}\}. \end{array}$$ (8)

For this joint distribution to be proper, the precision matrix *Q* = (**D**_*w*_−*ρ* **W**) needs to be invertible so that the covariance matrix **Σ** = *Q*^−1^ exists. This requires that the multiplicative parameter *ρ* to be in the interval (1/λ_*min*_, 1/λ_*max*_), where λ_*min*_ and λ_*max*_ are the smallest and the largest eigenvalues of the row-standardised spatial weights matrix, respectively (see Section 3.3.1 in [28, 39], p.250). The parameter *ρ* measures the strength of the spatial autocorrelation.

#### The Besag, York and Mollié (BYM) model

The Besag, York and Mollié (BYM) model [34] linearly combines the spatially-structured variability and the spatially unstructured variability to model the district-specific parameter *θ*_(*t*)*i*_ as

$$\begin{array}{c} \theta_{(t)i}=\beta_{0}+u_{(t)i}+v_{(t)i}, \end{array}$$ (9)

where *β*_0_ is the intercept, quantifying the average prevalence rate in the entire study region, i.e. Ethiopia, *v*_(*t*)*i*_ is the spatially-structured random component and *u*_(*t*)*i*_ the spatially-unstructured random component **u**_(*t*)_ = (*u*_(*t*)1_, …, *u*_(*t*)*m*_). The effects in the above model are district-specific and are modelled hierarchically. For the parameters in **v**_(*t*)_, the ICAR model is used and the parameters in **v**_(*t*)_ are modelled through an exchangeable model with *u*_(*t*)*i*_ independently $N(0,\sigma_{u}^{2})$ distributed for *i* = 1, …, *m*. Therefore, the BYM model is a convolution of an intrinsic CAR model and the independently identically distributed (IID) Gaussian model. Recall the sum-to-zero constraint on **v**_(*t*)_ in the ICAR model, and because of this constraint the prior mean of 0 for elements in vector **v**_(*t*)_ and the intercept *β*_0_ should be included to represent the overall level.

#### Leroux Conditional Autoregressive (LCAR) model

Since the BYM model is a convolution of an intrinsic CAR model and IID Gaussian model, it has an identification problem. As an alternative to the ICAR and the pCAR models, Leroux, Lei & Breslow [37] proposed a more general conditional autoregressive model (LCAR) in which the covariance structure of the model is a mixture of uncorrelated and correlated effects, specifically the LCAR has the following distribution

$$\begin{array}{c} v_{\left(t\right)}∼N(0,\sigma_{v}^{2}\;Q{\left(W,\rho \right)}^{-1}), \end{array}$$ (10)

where

Q(W,ρ)=ρW+(1-ρ)I,

and **I** is an identity matrix of order *m*, **W** is a neighborhood weight matrix, and the parameter *ρ* controls the overall degree to which the effects are correlated. If *ρ* = 0, then the LCAR model reduces to a model with independent random effects, and as in the pCAR model, it reduces to the ICAR model when *ρ* = 1. LCAR model allows the degree of clustering to be estimated, thereby circumventing having to use a BYM model with two components. Furthermore, if 0 ≤ *ρ* < 1, then the joint distribution of **v**_(*t*)_ with precision matrix *Q*(**W**, *ρ*) is proper [40].

#### Computation and models comparison

The classical approach to Bayesian inference is to use the Markov Chain Monte Carlo (MCMC) simulation techniques [41]. However, MCMC is computationally expensive. Hence, we used the integrated nested Laplace approximation (INLA) numerical method [42] to fit the Bayesian spatial models.

The spatial relationships of the districts were defined using a spatial weight matrix and neighbourhoods were defined using Queen’s contiguity whereby neighbours districts are sharing borders or a common vertex with each another. This was done using the nb2listw function in the spdep [42] R package. The Bayesian analyses were done using the inla() function in the R-INLA package [43]. The significance of the Moran’s *I* statistic was assessed using the Monte Carlo randomization technique.

The fitted models were compared using the Deviance Information Criterion (DIC) [44] and the widely applicable information criteria (WAIC) [45].

### Ethical consideration

Permission of the study was obtained from the School of Science Ethics Committee, University of South Africa (ERC Reference Number: 2021/CSET/SOS/045). In addition, permission to use the data for this study was obtained from Ethiopian Ministry of Health Office. In this study we have used aggregated district level data, therefore informed consent was not obtained from the study participants.

## Results

### Descriptive statistics

The total number of TB, HIV, and TB/HIV patients in Ethiopia stratified by gender is presented in Table 1. In 2018, 52,402 TB and 719,651 HIV patients were reported to the national Health Management Information System (HMIS) (Table 1). All these reported cases were considered in this study. Except for those districts where their TB or HIV data were not available, the data used covers all the regions, the zones and the districts of Ethiopia. Of a total number of 52,402 TB patienst enrolled in the Directly Observed Therapy Short Course (DOTS) and who were tested for HIV in Ethiopia in 2018, 55.32% (28,991) were male. However, a higher proportion of patients enrolled in HIV care who were screened for TB were female 62.91% (452,701).

**Table 1:** The number of TB, HIV and TB/HIV co-infected patients reported in Ethiopia by gender in 2018.

Variable	Male	Female	Total
Number of TB cases enrolled to DOTS and who were tested for HIV	**28,991**	**23,411**	**52,402**
Number of TB patients with HIV positive test result	**1,988**	**1,616**	**3,604**
Number of clients enrolled in HIV care who were screened for TB during their visit	**266,948**	**452,703**	**719,651**
Total number of HIV positive clients with active TB	**1,228**	**1,503**	**2,731**

HIV: Human Immunodeficiency Virus; TB: Tuberculosis; DOTS: Directly Observed Therapy, Short Course.

**Fig 2:**
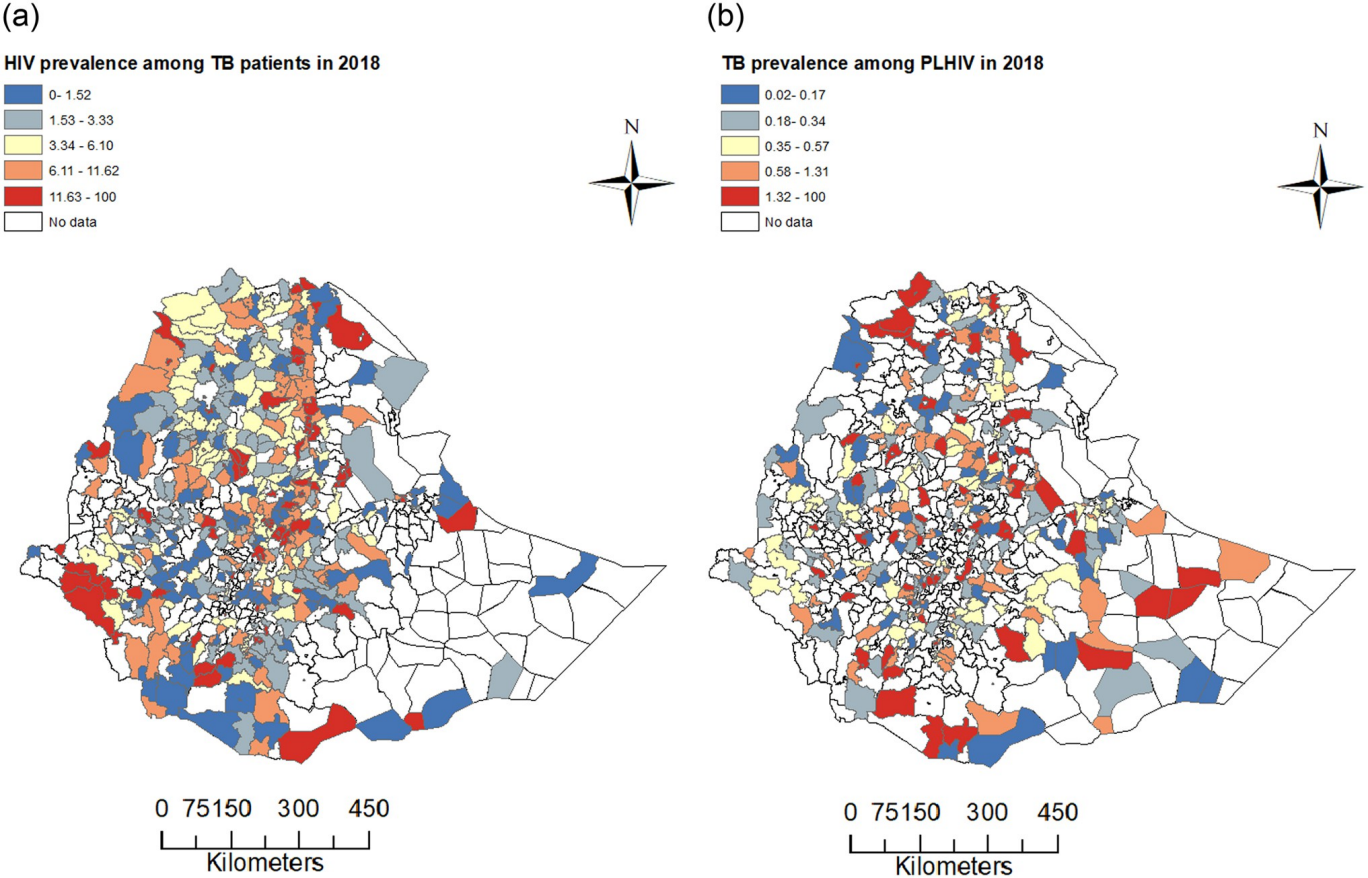
Prevalence plot. Geographical distribution of the prevalence of HIV among tuberculosis patients (a) and TB among PLHIV (b) in Ethiopia in 2018.

The prevalence of HIV among TB patients, denoted by HIV(TB), and the prevalence of TB among PLHIV, denoted by TB(HIV), in the regions and the two city administrations in 2018 are given in Table 2. The highest prevalence of TB among PLHIV was reported in the Gambella region (1.44%) and the overall prevalence of TB among PLHIV in Ethiopia in the same year was 0.38 percent (Table 2; Fig 2). On the other hand, the overall prevalence of HIV among TB patients in the country was 6.88 percent and the highest prevalence was observed in Addis Ababa (20.76%). The results in Table 2 and Fig 2 show that the prevalence of TB among PLHIV and that of HIV among TB patients were geographically heterogenous in 2018, and this will be further explored in the next sections. However, as the two maps in Fig 2 show, the observations that were made at a regional level are not necessarily reflected at all district levels within a region. For example, in Fig 2(b), except Harar, Gambella, and Dire Dawa city administration, there were some districts in other regions and Addis Ababa city administration which had high prevalences of TB among PLHIV.

**Table 2:** The prevalence of HIV among TB patients (HIV(TB)) and the prevalence of TB among PLHIV (TB(HIV)) by regions.

Region / City	HIV(TB)	TB(HIV)
Addis Ababa	20.76	0.44
Afar	5.08	1.42
Amhara	7.02	0.34
Beneshangul Gumuz	3.40	0.44
Dire Dawa	11.72	0.39
Gambela	9.72	1.44
Harari	13.06	0.25
Oromiya	3.98	0.31
SNNP	3.58	0.56
Somali	15.49	0.00
Tigray	8.00	0.63
Ethiopia	6.88	0.38

Two city administrations: Addis Ababa and Dire Dawa

### Spatial analyses

#### Spatial clustering of TB/HIV co-infection in Ethiopia

**Fig 3:**
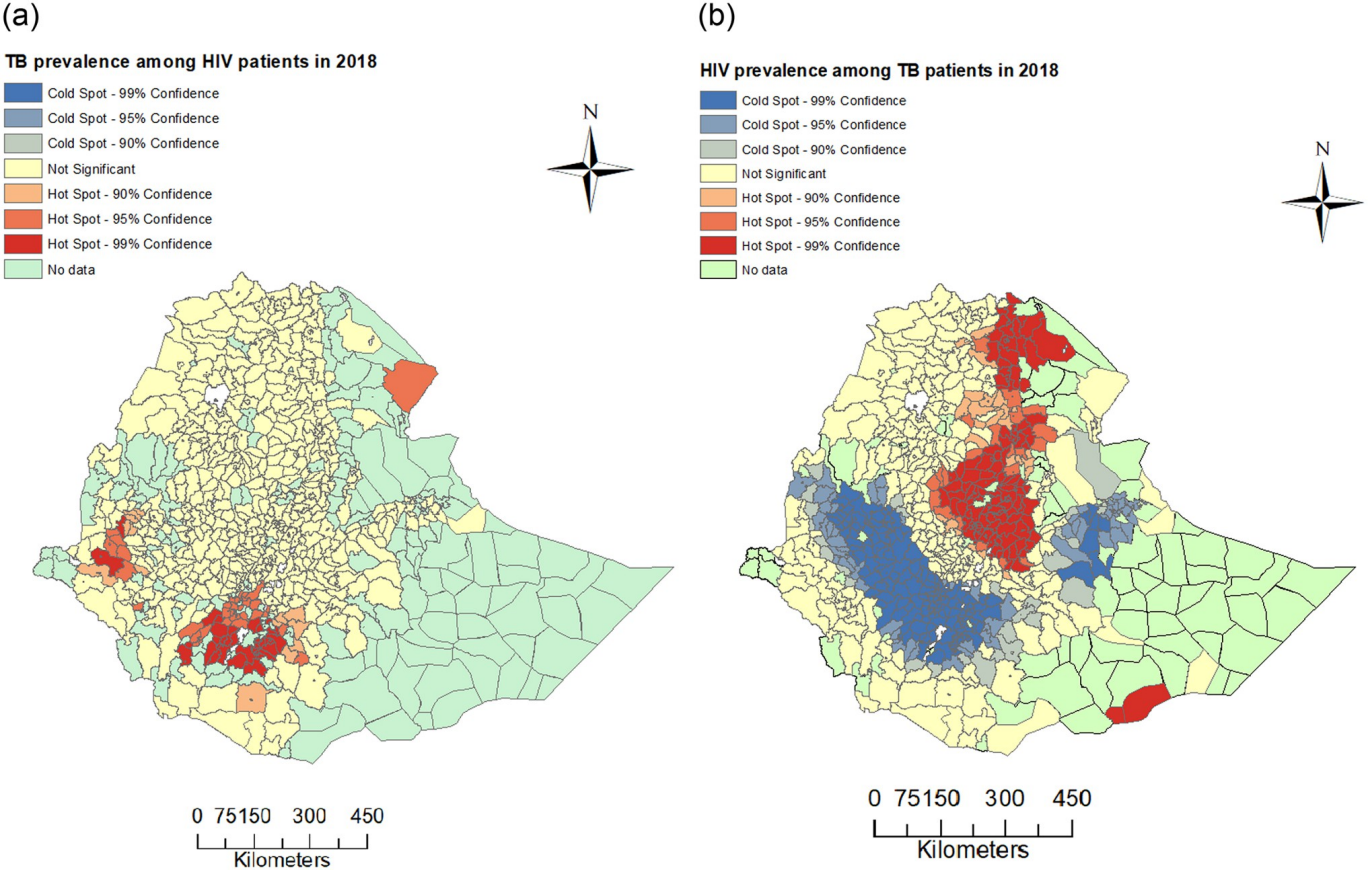
Spatial clustering plot. Spatial clustering for prevalence of TB among PLHIV (panel a) and HIV among TB patients (panel b) in Ethiopia at district level in 2018 using the Getis-Ord $G_{i}^{*}$ statistic.

There is evidence of spatial clustering of TB/HIV co-infection, specifically for HIV among TB patients (Global Moran’s *I* = 0.093, *p*-value = 0.0003). In the case of the prevalence of TB among PLHIV, the Global Moran’s *I* = 0.017 and is statistically nonsignificant with *p*-value = 0.2663. The positive Global Moran’s *I* values show that any two spatial neighbouring districts tend to have a similar prevalence of TB among PLHIV and similar prevalence of HIV among TB patients. To explore the spatial heterogeneity further and identify the hot-spots and cold-spots districts for the prevalence of both HIV among TB patients and TB among PLHIV, we used the Getis-Ord $G_{i}^{*}$ statistic. Maps showing the distribution of the spatial clusters of the prevalence are presented in Fig 3. Fig 3(a) shows that the hot-spots were: at the border of Awsi Rasu zone of Afar with Djibouti, the districts of Qeleme Wellega zone in the Oromia region; in the districts of Gamo Gofa, South Omo, Wolaita, Dawuro and Gedio zones in SNNP region. There was no cold-spot. Fig 3(b) shows that hot-spots for the prevalence of HIV among TB patients in Ethiopia were: in the districts located in Afar, Amhara, Oromiya, Somali and Tigray regions and further in Addis Ababa city administration. However, the cold-spots for the prevalence of HIV among TB patients were concentrated in the districts of Benishangul-Gumuz region; Wellega, Illu Aba Bora and Jimma zones of Oromia region; Harer region; districts of Kefa, Konta, and Gamo Gofa zone in SNNP regions; and Dire Dawa city administration.

### Bayesian spatial modeling for TB/HIV co-infection

Since the global Moran’s *I* statistic values for both prevalence data were positive, we considered fitting the Bayesian spatial logistic models to assess the amount of variability in each co-infection type that is explained by the variations between spatially structured random effects. The Bayesian approach requires the specification of the prior distributions for all the random elements of the model. Since the empirical information on the parameters, *β*_0_, *σ*_*v*_, and *σ*_*u*_ or relevant to the study data was not available, non-informative priors were used [46]. Specifically, for the intercept a non-informative prior *N*(0, 1000) was used. For *σ*_*v*_ and *σ*_*u*_ in the four priors or models ICAR, pCAR, BYM and LCAR, the default minimally informative priors that are specified in the log of the structured and unstructured effect precision parameters, i.e. *log*(*τ*_*v*_)∼*logGamma*(1, 0.0005) and *log*(*τ*_*u*_)∼*logGamma*(1, 0.0005) [47] were used.

Table 3 displays the model selection criteria *DIC* and *WAIC* from fitting the above four prior models to the data. The DIC and WAIC values for the BYM prior were smaller than the other priors except for TB among PLHIV data, where the values are slightly smaller for LCAR model. Hence the BYM prior is the preferred prior for both data sets. Therefore, in what follows, we only report results based on BYM prior outcomes for the data.

**Table 3:** Summary of DIC and WAIC values for different priors used to fit the Bayesian spatial models on prevalence of HIV among TB patients, and TB among PLHIV.

	Models comparison criteria			
	HIV among TB patients		TB among PLHIV	
Prior	DIC	WAIC	DIC	WAIC
iCAR	2544.307	2527.077	1940.760	1967.126
pCAR	2528.100	2470.864	1906.281	1913.731
BYM	2506.427	2445.771	1898.015	1903.040
LCAR	2533.809	2467.982	1898.649	1904.488

**Fig 4:**
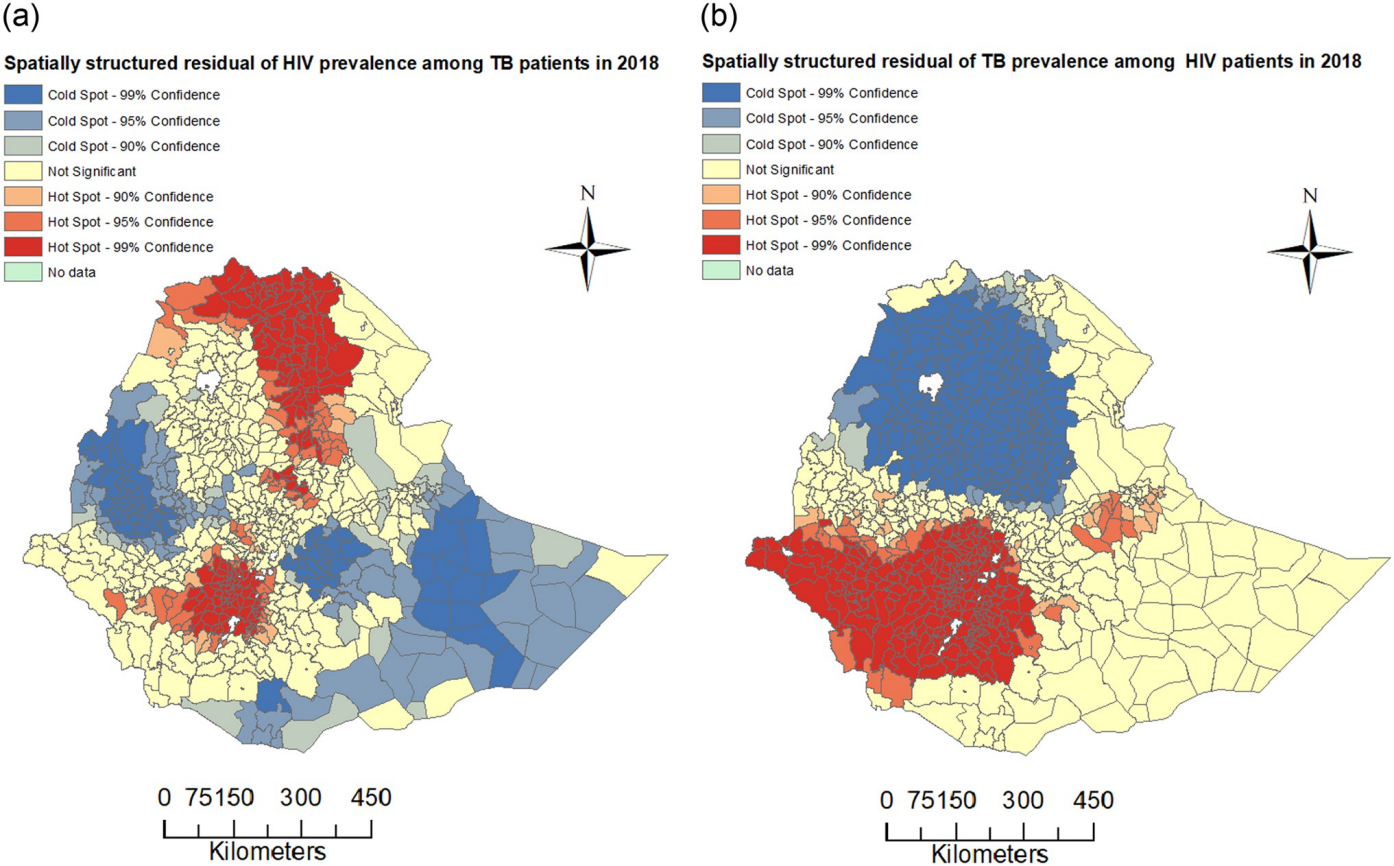
Spatial residual plot. Spatial clustering of spatially structured residuals from fitted BYM models to HIV among TB patients and TB among PLHIV data using the Getis-Ord $G_{i}^{*}$ statistic.

The spatially structured residuals of the fitted BYM model were examined for spatial autocorrelation. The global Moran’s *I* for spatially structured residuals was 0.350 (with *p*-value <0.001) for HIV among TB data and 0.596 (with *p*-value <0.001) suggesting that there was strong spatial autocorrelation in each of the residuals data. The maps for spatial clustering of these residuals are given in Fig 4. The plot in panel (a) shows that there are districts that were identified as hot-spots in the Gambella region, in most of the western areas and some parts of the west Hararge zone of the Oromia region, and the SNNP region for spatially structured residuals from BYM model fitted to the prevalence of TB among PLHIV data. The plot of spatially structured residual predicted from BYM model by fitting the prevalence of HIV among TB patients data shows hot-spot areas (districts) in Tigray region, northwest, southwest, and west Afar areas, border of north Gondar and Sudan, districts in north and south Wollo zones in Amhara region, south part of Jimma zone and Finfine Zuria of Oromia region, districts in Kefa, Wolayta, Hadiya and south Omo zones of SNNP region. The hot-spots show that there was spatial clustering of residuals, which agrees with the global Moran’s *I* statistic values. These results indicate that district-level heterogeneity in the prevalence of HIV among TB patients and TB among PLHIV in Ethiopia in 2018 was not fully explained by the two random effects for both data.

The variances of spatially structured random effect and unstructured effect of BYM model fitted to HIV among TB patients data were ${\hat{\sigma }}_{v}^{2}=2.469$ and ${\hat{\sigma }}_{u}^{2}=0.968$, respectively, while variances of BYM model fitted to TB among PLHIV were ${\hat{\sigma }}_{v}^{2}=19.032$ and ${\hat{\sigma }}_{u}^{2}=0.713$, respectively. Therefore, about 71.8% and 96.4% of the variability in the prevalence of HIV among TB patients and TB among PLHIV were explained by the spatial structured random effects, respectively.

## Discussion

TB and HIV still represent the most important infectious diseases in the globe despite the measures taken by WHO to eradicate them. These diseases still present alarming data on morbidity and mortality [1, 48]. In this study, we have investigated the spatial clustering and heterogeneity in the prevalence of TB/HIV co-infection, i.e. the prevalence of TB among PLHIV and HIV among TB patients in Ethiopia at the district level for 2018.

The prevalence of HIV among TB patients and TB among PLHIV varied between regions and between districts in Ethiopia. The findings of the study show that the prevalence of the TB/HIV co-infection (TB among PLHIV and HIV among TB patients) was geographically heterogeneous. These results are consistent with the findings of [14], whose results indicate that the prevalence of TB among PLHIV and the prevalence of HIV among TB patients varied in Ethiopia at the district level but the data that we have used was yearly aggregated data for 2018 but in their study, they have used three years aggregated data.

The results from LISA using Getis-Ord $G_{i}^{*}$ statistics showed that the prevalence of TB among PLHIV was spatially clustered, i.e. there were hot spots in districts of Qeleme Wellega zone in the Oromia region and districts of Gamo Gofa, South Omo, Wolayta, Dawuro, and Gedio zones in the SNNP region. However, there was no cold spot observed.

Findings from similar studies conducted in Ethiopia [13, 14] revealed educational status, wealth index, migrations, drug dependency, WHO clinical stage, and baseline CD4 count as significant predictors of this TB/HIV co-infection. Additionally, the results of other related studies conducted in several countries identified poverty, poor healthcare, drug, and alcohol misuse, and mental disorders as the main determinant factors for the spatial variation of TB/HIV co-infection [14, 17, 49, 50].

In this study, we have noticed that the hot spots for the prevalence of TB among PLHIV have appeared in the country more in urbanized areas such as Awassa, Jimma, Shashemene, Hosaena (Hadiya zone), and Sodo town (in Wolayta zone). This may be related to the migration of people within districts or across neighboring districts for a job and better living conditions. Studies in Ethiopia have indicated that spatial clustering of TB was associated with migration [49, 51–55] and ongoing TB transmission is also high in overcrowded and congested urban areas [49, 52, 55, 56].

Some studies conducted in Brazil [50, 57, 58] also showed that strong spatial clustering of TB and TB/HIV observed in urban areas and semi-urban areas with a high population density and market places.

The LISA cluster maps also illustrate that there was a hot-spot area on the border of the Awsi Rasu zone of Afar with Djibouti. This finding supports the results of the existing literature [14, 55], therefore it may affirm that there is a relationship between TB transmission and international border or territorial space. Hence it is necessary to extend the country-level analysis to higher spatial dimensions that include at least neighbouring countries to obtain global solutions and targeted intervention [59–61].

Further, there were districts identified as hot-spots for the prevalence of HIV among TB patients in Afar, Amhara, Oromiya, Somali (at the border of Dolobay and Somalia in the southeastern part of Ethiopia), and Tigray regions and further in Addis Ababa city administration. This spatial variability is consistent with the results from [14] who used three years data and found clustering of HIV among TB patients in districts located in Afar and Amhara regions.

However, unlike the prevalence of TB among PLHIV data, some districts or areas were identified as the cold-spots for the prevalence of HIV among TB patients in the Benishangul-Gumuz region, Wellega, Illu Aba Bora, and Jimma zones of Oromia region, Harer region, districts of Kefa, Konta, and Gamo Gofa zones in SNNP regions, and Dire Dawa city administration. Hot-spot areas for HIV among TB patients were also observed at the border of Adigrat (in Tigray region) and Eritrea, and Dolobay area (in Somali region) and Somali. There is a refugee camp close to the border between Adigrat and Eritrea. Generally, refugees are often in situations where they do not have proper job access to generate additional income. As a result, women and young girls in refugee camps often enter commercial sex work to earn income for food, and to gain access to other resources [62], this exposes them to HIV/AIDS and other sexually transmitted diseases (STDs) infections. Further, in these areas, HIV vulnerability can raise as districts have poor access to health care facilities and integrated service provision to address TB/HIV co-infection [14]. The current study results revealed that the prevalence of HIV among TB patients was more spatially correlated than the prevalence of TB among PLHIV in Ethiopia. This finding agrees with Aturinde et al. [63] who found that HIV was more spatially correlated than TB in Uganda for the period 2015 to 2017. The positive global Moran’s *I* statistic values for both data were suggesting that neighbouring districts tend to possess similar characteristics in the prevalence of TB/HIV co-infection.

The results from spatial clustering analyses on the spatially structured residuals obtained from fitting the Bayesian spatial logistic regression with BYM priors suggested that for the study data, not all the district-level heterogeneity in the prevalence of HIV among TB patients and in the prevalence of TB among PLHIV in Ethiopia for 2018 were explained by the spatially structured random effects.

Unlike the results of the current study, study conducted in Kenya [64], which applied Bayesian hierarchical approach to study the spatial distribution of tuberculosis reported that spatially unstructured model had better fit for their study data.

In addition, the hot-spots districts may indicate an expanded epidemiological view of the districts with a high risk of TB/HIV co-infection in the country. These spatial heterogeneities in the prevalence indicate an imperative for more regular surveillance to detect emerging and existing TB/HIV hot-spots areas for targeted health interventions by authorities to prevent TB and HIV transmissions, especially in settings where there are limited resources.

There were some limitations to this study that could have affected our findings. First, the data were aggregated at the district level, therefore the findings of this study cannot be representative of small administrative units of the country or Kebele or household or individual level. Second, since the data were collected from the national HMIS electronic surveillance system, the notified HIV and TB cases might not reflect the actual burden of the diseases in a district due to underreporting of cases or miss detection. For example, symptomatic individuals who did not access HIV or/and TB diagnosis and treatment might remain unreported.

## Conclusion

In this paper, we assessed the spatial clustering and heterogeneity of the prevalence of two types of TB/HIV co-infection applying the global Moran’s *I* statistic, Getis-Ord $G_{i}^{*}$ statistic with LISA cluster maps, and fitting the Bayesian spatial models. The results applying these statistical methods show that the prevalence of TB among PLHIV and HIV among TB patients were strongly spatially clustered in Ethiopia at the district level in 2018. The LISA detected distracts or areas that were hot-spots and cold-spots for TB among PLHIV and HIV among TB patients in various regions and the two city administrations. The analysis of spatially structured and unstructured random effects of Bayesian spatial modeling showed that most of the variability in each of the two prevalence was explained by the spatially structured random effects. This study expanded knowledge about the clustering of TB/HIV co-infection in Ethiopia at the district level. The findings provide information to health policymakers in Ethiopia to plan geographically targeted and integrated interventions to jointly control TB and HIV.

## Abbreviations

AIDS: Acquired immunodeficiency syndrome
DOTS: Directly Observed Therapy, Short Course
HMIS: Health Management Information System
HIV: Human Immunodeficiency Virus
LISA: local indicators of spatial analysis
STDs: Sexually transmitted diseases
